# Meningitis Risk in Patients with Inner Ear Malformations after Cochlear Implants: A Systematic Review and Meta-Analysis

**DOI:** 10.1097/MAO.0000000000003913

**Published:** 2023-06-15

**Authors:** Shravan Gowrishankar, Alex Fleet, Michele Tomasoni, Isla Kuhn, James Tysome, Matthew E. Smith, Neil Donnelly, Patrick Axon, Daniele Borsetto, Manohar Bance

**Affiliations:** ∗ School of Clinical Medicine, University of Cambridge, Cambridge, UK; † Department of Otolaryngology—Head and Neck Surgery, Department of Medical and Surgical Specialties, Radiological Sciences and Public Health, University of Brescia, Brescia, Italy; ‡ Cambridge University Medical Library; § Department of Otolaryngology—Head and Neck Surgery, Cambridge University Hospitals NHS Foundation Trust, Cambridge, UK

**Keywords:** Cochlear implant, Common cavity, Complication, IEA, IEM, Incomplete partition, Inner ear malformation, Meningitis, Mondini, Postoperative complication, Postoperative meningitis, Surgical complication

## Abstract

The objective of this study is to determine the rate of postoperative meningitis after cochlear implantation in those with inner ear malformations (IEMs) via meta-analysis.

**Data sources:**

Medline, EMBASE, and the Cochrane Library.

**Methods:**

This study was reported following the preferred reporting items for systematic reviews and meta-analyses (PRISMA) checklist. Proportion meta-analysis was conducted through an inverse variance random-effect model based on arcsin transformation and presented as forest plots. Quality assessment of the included studies was performed through the National Institutes of Health Quality Assessment Tool.

**Results:**

Overall, 38 of 2966 studies met the inclusion criteria and were included in the analysis. There were 10 cases of meningitis after cochlear implantation in 1300 malformed ears. The overall rate of meningitis after cochlear implantation in IEMs was 0.12% (95% confidence interval, 0.006–0.380%; *I*^2^ = 0%). Cases occurred in incomplete partition (n = 5), Mondini deformity (n = 2), common cavity (n = 2), and enlarged internal auditory canal (n = 1). Six of 10 cases of postoperative meningitis occurred with an intraoperative cerebrospinal fluid leak.

**Conclusion:**

In those with IEMs, the risk of meningitis after cochlear implantation is very low.

## INTRODUCTION

Cochlear implants (CIs) are surgically implanted devices that can improve hearing in those with severe to profound sensorineural hearing loss. By restoring sound perception, they also have wider effects on quality of life by reducing social isolation, anxiety, and depression in implanted patients (1).

However, as with any surgical intervention, there are potential complications associated with CIs. Minor complications often resolve spontaneously or with medical management, and range from dizziness to taste disturbance. Major complications may require revision surgery and/or hospitalization, and include device migration, electrode extrusion, and infections such as meningitis (2).

Meningitis is a rare but life-threatening complication linked to CIs that has received a significant level of attention. This concern stemmed from a large epidemiologic study in the early 2000s that found a higher risk of meningitis in those with CIs compared with the general population (3). In those receiving cochlear implantation, proposed risk factors include CIs with intracochlear positioners. However, the cause for this is not entirely known, with several etiologies being proposed, one of which includes positioner-induced modiolus trauma. Another proposed risk factor for postoperative meningitis includes the presence of inner ear malformations (IEMs) (3–5).

IEMs can be classified into categories based on morphology, which may arise from developmental arrest, genetic abnormalities, or intrauterine factors (6,7). Abnormalities during early development can lead to the cochlea being completely absent (*cochlear aplasia)* or underdeveloped (*cochlear hypoplasia*). A cystic structure can result if differentiation between the cochlea and vestibule does not proceed (*common cavity).* Even if differentiation is successful, disruption of the central modiolus and/or interscalar septa can result in a cystic structure internally (*incomplete partition).* Other abnormalities include an *enlarged vestibular aqueduct* (EVA) or dysplasia/aplasia of the vestibular system and cochlear nerve. These malformations can also occur in combination, as is the case in *Mondini deformity.* This consists of the triad of incomplete partition II, dilated vestibule, and EVA (7). However, the term has been used to describe a range of other IEMs (8).

There are a range of reasons why IEMs could be at increased risk of meningitis. The presence of abnormal fistulae between the inner ear and subarachnoid space could allow communication and infection spread to the cerebrospinal fluid (CSF) (9). Other reasons include malformations of the lamina cribrosa, which is a bony separation between the cochlea and internal auditory canal (IAC). This may be partially or completely absent and allow a route of infection from the middle ear to the inner ear and the CSF space, leading to a higher potential for otogenic meningitis (10). The CI can act as a nidus for infection, facilitating infection spread.

Many studies that have proposed a link between IEMs and meningitis after cochlear implantation have been case reports or small case series, making it difficult to gauge true incidence and risk (11,12). The aim of this study is to perform a systematic review and proportion meta-analysis of the literature to ascertain the rate of meningitis after CIs in those with IEMs.

## METHODS

This systematic review and meta-analysis were performed in line with the preferred reporting items for systematic reviews and meta-analyses (PRISMA) guidelines (13). This review was also registered on PROSPERO (Registration ID: CRD42022333508).

### Aims

The aim was to perform a proportion meta-analysis of the prevalence of postoperative meningitis in patients with IEMs after cochlear implantation. The specific type of IEM implanted was recorded to determine whether a specific IEM was prone to a higher risk of meningitis.

### Search Strategy

The search strategy was designed with assistance from our University Medical librarian (I. K.). A search on Medline, EMBASE, and the Cochrane Library was performed in January 2023, and word variants were combined from two key themes: (A) CIs and (B) IEMs (Supplementary File 1, http://links.lww.com/MAO/B656). This led to 966 unique hits.

A second search was also performed combining “cochlear implantation” and “complications” (Supplementary File 1, http://links.lww.com/MAO/B656). This was designed to capture relevant studies that reported complications after implantation in a mixed population of patients: some with normal cochleae and others with IEMs. These studies fit the inclusion criteria as it was possible to determine whether meningitis, if reported, occurred in IEM or normal cochlea patients (14–16). However, some of these studies were missed by the first search. This was potentially because they were only indexed under “cochlear implants” and not under “IEMs” as the latter were only a minor component of the article. This search yielded 2000 unique hits.

Results from both searches were combined to increase comprehensiveness, yielding 2,966 original articles. Two authors (S. G. and A. F.) independently screened all the titles and abstracts resulting from the search, and then assessed the full texts of the relevant articles identified against the inclusion criteria. Disagreements were resolved through discussion. A third author (D. B.) resolved disagreements if discussion failed to reach a consensus. The references of all narrative reviews found were also screened to find relevant articles.

### Inclusion and Exclusion Criteria

The inclusion criteria are presented through a PICOTS format in Table 1. The exclusion criteria included non-English language studies, case reports, editorials, letters, and reviews. Studies reporting other otologic surgery at the same time as cochlear implantation were also excluded.

**TABLE 1:** Description of the study design using the PICOTS format (also containing the inclusion criteria for the study)

Domain	Description
Population and intervention	Human individuals of any age with IEMs undergoing cochlear implantation with any device.
Comparison	Not applicable.
Outcome	Meningitis occurring in the postoperative period.
Time	Meningitis recorded at any time postoperatively was included; no cut-offs were placed on minimum follow-up time. Studies published in any year were included, with no cut-offs by year.
Study type	Any study design reporting a cohort of patients with IEMs undergoing cochlear implantation and tracking postoperative complications were included in our systematic review and meta-analysis.

### Data Extraction

An electronic data collection form was used to collect the following information from included studies: author, year of publication, study design, number of patients with IEMs, sex breakdown, mean age at implantation, number of malformed ears implanted, radiologic confirmation of IEMs with high resolution CT scan and/or MRI, types of malformed ears implanted, number of postoperative cases of meningitis, number of intraoperative CSF leak (including gusher), and postoperative CSF leak and follow-up time (mean and range). The categories of IEMs were recorded according to Sennaroglu's version of the modified Jackler classification (6,7) and included cochlear aplasia, cochlear hypoplasia, common cavity, and incomplete partition of the cochlea (split into types I, II and III if specified [7]), and EVA. Cochlear ossification was not included. Mondini deformity was recorded if the complete triad of incomplete partition II, minimally dilated vestibule, and an EVA were present, or if the study reported Mondini dysplasia unspecified.

The data extraction form was designed by S. G., D. B., and M. B. Two reviewers independently extracted the data, and disagreements were resolved by a third reviewer. When relevant conference abstracts were identified, the authors were contacted to obtain data on the number of postoperative meningitis cases in their IEM CI cohort.

### Statistical Analysis

Proportion meta-analysis was conducted through an inverse variance random-effect model based on arcsin transformation and presented as forest plots. The weighted pooled proportion estimates and corresponding 95% confidence intervals were calculated according to the random-effects models of DerSimonian and Laird (17). For each study, proportions are depicted as gray squares, whereas relative 95% confidence interval as horizontal lines. The weight of each study on the overall effect estimate is reported and represented by the square size. The overall proportion estimates with relative 95% confidence intervals are depicted as black diamonds at the bottom of the forest plot. Heterogeneity between studies was assessed with Higgins *I*^2^ and *τ*^2^ tests, defined as low if *I*^2^ < 25%, moderate if between 25–50%, and substantial if >50% (18). Publication bias was assessed through funnel plots and Egger's test (19,20). Moderator analysis was conducted through subgroup analysis to assess whether the year of publication and number of patients included could be related to a higher risk of postoperative meningitis (19,21). Statistical analysis was performed with R (version 4.2.1, R Foundation for Statistical Computing, Vienna, Austria), packages “meta” and “metafor.” Statistical significance was defined as *p* < 0.05.

### Risk of Bias Assessment

The National Institutes of Health Quality Assessment Tool was used to analyze each study for risk of bias. Bias analysis was performed by two reviewers independently (S. V. G. and A. F.), and disagreements were resolved through a third reviewer (D. B.).

## RESULTS

### Literature Search

**FIG. 1:**
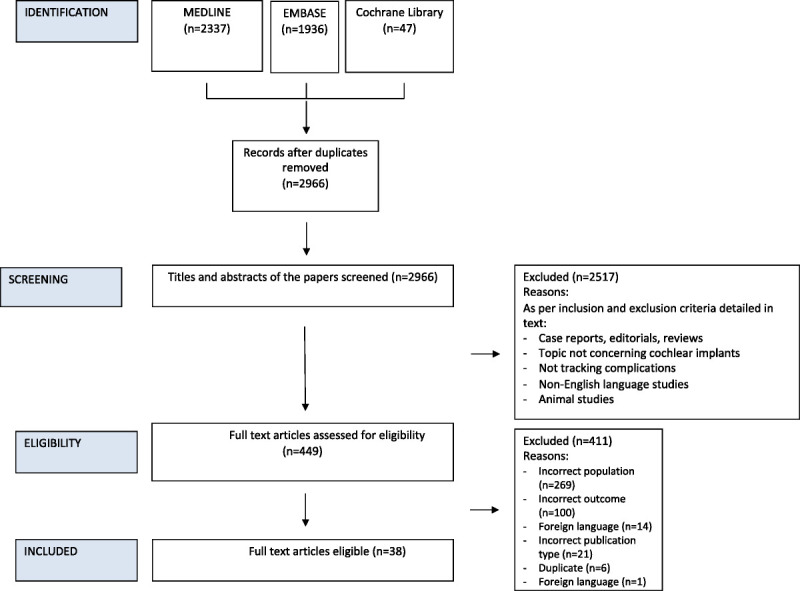
PRISMA flowchart of the search and screening process.

The first search specifying IEMs produced 966 unique hits. The second, broader search, with “CIs” and “complications” produced 2000 unique hits. Reasons for these separate searches are outlined in the methods. The results of both searches were combined to yield 2,966 unique hits. After screening these via title and abstract, 449 articles remained for full text screening. From this set, 38 articles were included in the final review after applying the inclusion and exclusion criteria. This is shown via a PRISMA flowchart in Figure 1

### Background Characteristics

The characteristics of all the included studies are outlined in Table 2 (5,12,14–16,22–54). All 38 studies were descriptive cohort studies without control (55). Thirty-one studies provided the mean age at implantation. Thirty of these studies reported a mean age of <18 years, with 18 of these 30 studies reporting a mean age of <5 years. Six studies reported on the pneumococcal vaccination status of the population before implantation, confirming that all were vaccinated.

**TABLE 2:** Characteristics of included studies

Authors	Year of Publication	Study Design	Number of Patients	Number of Implanted Ears	Mean Patient Age in Years	M/F	Cases of Meningitis	Vaccination Status	Mean Follow-up Length in Years (Range)
Ahn et al.	2008	Descriptive cohort	73*^a^*	73*^a^*	10.7	N/A	0	N/A	3.4 (0.6–6.6)
Ahn et al.	2011	Descriptive cohort	11	11	4.5	5/6	2	N/A	6.7 (4.4–10.4)
Bae et al.	2022	Descriptive cohort	8	10	4.9	4/4	0	N/A	3 (1.5–5)
Bajin et al.	2018	Descriptive cohort	73	76	11.4	41/32	2	N/A	2.5 (0.5–15)
Beltrame et al.	2013	Descriptive longitudinal cohort	19	19	3.4	12/7	0	All vaccinated against meningitis	(2–5)
Bent et al.	1999	Descriptive cohort	10	10	7.8	6/4	0	N/A	1.2 (0.5–3)
Berrettini et al.	2013	Descriptive case series	4	4	3.4	1/3	0	All vaccinated against *Pneumococcus* and *Haemophilus influenzae*	1.6 (1–2.5)
Eftekharian et al.	2019	Descriptive cohort	18	18	7.1	7/11	0	N/A	5.5 (3.3–9)
Grover et al.	2021	Descriptive cohort	25	25	4	N/A	0	N/A	2
Halawani et al.	2020	Descriptive cohort	32	32	4	N/A	0	N/A	3 (0.75–6)
Kim et al.	2006	Descriptive cohort	46	46	5.9	27/19	0	N/A	N/A
Kontorinis et al.	2012	Descriptive cohort	33	39	9.2	12/21	0	N/A	11.8 (1–17)
Lai et al.	2012	Descriptive cohort	12	12	2.4	7/5	0	N/A	N/A
Lee et al.	2010	Descriptive cohort	23	27	5.3	11/12	0	N/A	Mean not provided, minimum follow-up was 2 yr
Lescanne et al.	2011	Descriptive cohort	19	19	N/A	N/A	0	All received pneumococcal vaccination preoperatively, then every 5 yr postoperatively	(0.5–15)
Li et al.	2014	Descriptive cohort	47	47	N/A	N/A	0	N/A	0.1
Loundon et al.	2008	Interventional cohort	37	37	8.1	N/A	1	All received pneumococcal vaccination	3.9 (0.1–15)
Luntz et al.	1997	Descriptive cohort	10	10	6.5	N/A	0	N/A	2.4
Manzoor et al.	2016	Descriptive cohort	17	32	6.8	10/7	0	N/A	4.2 (0.2–13.1)
Mey et al.	2016	Descriptive cohort	55	80	23.8	22/33	0	All received preoperatively pneumococcal vaccination	N/A
Mylanus et al.	2004	Interventional cohort	13	13	4.4	N/A	0	N/A	3.5 (1–9)
Pradhananga et al.	2015	Descriptive cohort	5	5	2.8	1/4	0	N/A	Mean not provided, minimum follow-up was 3 yr
Qi et al.	2019	Descriptive case series	108	108	1.6	57/51	0	N/A	5
Rachovitsas et al.	2012	Descriptive cohort	6	6	6.6	5/1	1	N/A	3.5 (1.9–10)
Sharma et al.	2021	Descriptive cohort	24	24	3.6	N/A	0	N/A	2
Smeds et al.	2017	Descriptive cohort	10	15	1.8	9/1	0	All received preoperative *H. influenzae* type B and pneumococcal vaccines	4.2 (0.1–8.1)
Suk et al.	2014	Descriptive cohort	23	25	5.3	14/9	0	N/A	4.7 (1.1–11.2)
Suri et al.	2021	Descriptive cohort	27	27	N/A	N/A	0	N/A	3
Tarkan et al.	2013	Descriptive cohort	17	17	N/A	N/A	2	All received pneumococcus vaccination preoperatively, then every 5 yr postoperatively	4.8 (0.5–12)
Tay et al.	2019	Descriptive cohort	20	25	3.3	10/10	0	N/A	N/A
Theunisse et al.	2018	Descriptive cohort	Not stated*^b^*	87	N/A	N/A	2	N/A	7.9 (0.1–27.2)
Tian et al.	2018	Descriptive cohort	14	14	3.7	8/6	0	N/A	1
Van Wermeskerken et al.	2007	Descriptive cohort	9	9	3.9	5/4	0	N/A	1.9 (0.5–4)
Wei et al.	2017	Interventional cohort	13	13	4.8	5/8	0	N/A	(0.25–2)
Yang et al.	2020	Descriptive cohort	16	19	<1*^c^*	N/A	0	N/A	(1–2)
Xia et al.	2015	Interventional cohort	21	21	4	14/7	0	N/A	3
Ding et al.	2009	Descriptive cohort	229	238	N/A	N/A	0	N/A	N/A
Gysin et al.	2000	Descriptive cohort	7	7	N/A	N/A	0	N/A	Up to 8

*^a^*Common cavity cases were excluded from this study due to population overlap from a later article (Ahn et al., 2011).

*^b^*The corresponding authors were contacted to retrieve these data without success.

*^c^*All patients were younger than 12 months, but an average age at implantation was not provided.

CI indicates cochlear implant; IEM, inner ear malformation; N/A, information was not provided or tracked in the study.

### Number and Breakdown of Malformations

CIs were placed in a total of 1,300 ears with IEMs. The most common malformation implanted was EVA (n = 332), followed by Mondini deformity (n = 297) and incomplete partition of the cochlea only (n = 158). Incomplete partition in combination with EVA was recorded in 56 cases. The least common malformation was cochlear aplasia (n = 1). The underlying abnormality was not provided for 196 ears. A full breakdown by the type of malformation is given in Table 3. One hundred thirty-one ears had other abnormalities outside the main categories of Sennaroglu's classification (7), such as semicircular canal dysplasia, and other vestibular defects.

**TABLE 3:** Breakdown of malformed ears by the type of IEM and the number of postoperative meningitis cases by type of IEM

Type of IEM	Numbers Implanted	Number of Cases of Postoperative Meningitis
Cochlear aplasia	1	0
Cochlear hypoplasia	11	0
Common cavity	118	2
Incomplete partition only	158	5
(a) Type I	51	2
(b) Type II	47	1
(c) Type III	29	0
(d) Unspecified	31	2
Incomplete partition + EVA	56	0
EVA only	332	0
Mondini deformity	297	2
Other abnormalities	131	1
Unspecified	196	0
Total	1,300	10

EVA indicates enlarged vestibular aqueduct; IEM, inner ear malformation.

Of note, 297 implanted ears had Mondini deformity. Of these, 217 were documented as having the triad of incomplete partition type 2, dilated vestibule, and EVA. We found that 80 ears were classified as Mondini, but the underlying deformities were not specified. Because of the discrepancies with this term, it is possible other abnormalities could have been classified as Mondini in these 80 cases.

### Number of Meningitis Cases

**FIG. 2:**
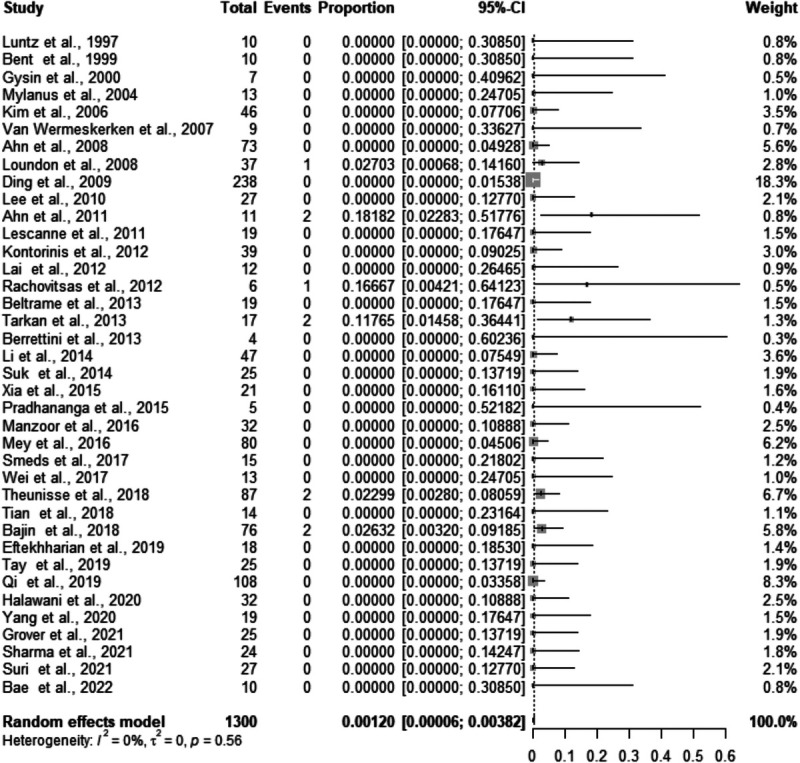
Forest plot for studies reporting the rate of postimplant meningitis in those with IEMs.

A total of 10 cases of meningitis were recorded in 1300 ears implanted with IEMs. The meta-analysis returned a rate of meningitis after CI in IEM of 0.12% (95% confidence interval, 0.006–0.382%; *I*^2^ = 0%) (Fig. 2). No publication bias was found with Egger's test (*p* = 0.515).

Cases were recorded in those with incomplete partition only (n = 5), Mondini deformity (n = 2), common cavity (n = 2), and enlarged IAC (n = 1). For each case of postoperative meningitis reported (n = 10), available data on demographics, intraoperative factors such as electrode insertion technique, CSF leak, vaccination status, and causative organism are provided in Table 4.

**TABLE 4:** Characteristics of those with postoperative meningitis

Case Number	Age (Yr) and Sex	Type of Malformation	Electrode Insertion Technique	Electrode Type	Presence of a Positioner	Time from Implantation to Meningitis	Vaccination Status	Causative Organism	Treatment	Intraoperative CSF Leak
1 (Ahn et al., 2011)	5.2, M	Common cavity	Cochleostomy	Advanced Bionics (Clarion High Focus 1.2)	N/A	N/A	N/A	N/A	Had to undergo reimplantation because of recurrent meningitis and device failure. No meningitis reported after second implant (5-yr follow-up)	Yes
2 (Ahn et al., 2011)	3.8, F	Common cavity	Cochleostomy	Cochlear (Nucleus CI24RST)	No	N/A	N/A	N/A	N/A	No
3 (Loudon et al., 2008)	3, M	Incomplete partition (type unspecified)	Cochleostomy	N/A	N/A	5 d	N/A	Bacterial meningitis—no clarifying details	Medical treatment with antibiotics led to complete resolution	No
4 (Tarkan et al., 2013)	2.5, F	Incomplete partition type I	Round window	Med-El (Sonata)	No	30 mo	Pneumococcal vaccination preoperatively	No bacterial growth identified on CSF culture	Had recurrent meningitis treated with antibiotics. Revision surgery and reimplantation performed. Follow-up after reimplantation not provided.	Yes—sealed with temporalis fascia
5 (Tarkan et al., 2013)	1.5 (sex unavailable)	Incomplete partition type I	Round window	Cochlear (Nucleus CI 24 RE)	No	1 mo	Pneumococcal vaccination preoperatively	N/A	Resolution after 15 d of antibiotic treatment. No recurrence as of 17 mo follow-up	Yes—sealed with temporalis fascia
6 (Theunisse et al., 2018)	N/A	Incomplete partition type II	N/A	N/A	N/A	N/A	N/A	N/A	N/A	No
7 (Theunisse et al., 2018)	N/A	Enlarged IAC	N/A	N/A	N/A	N/A	N/A	N/A	N/A	No
8 (Rachovitsas et al., 2012)	4.5, F	Incomplete partition (unspecified)	Cochleostomy	Cochlear (Contour Freedom)	No	In the following days (exact time unspecified)	N/A	N/A	Removal of implant and medical management	Yes—sealed with temporalis fascia and tissue
9 (Bajin et al., 2018)	N/A	Mondini	Cochleostomy	Med-El (Form 19)	No	In the first month	N/A	N/A	Intravenous antibiotics	Yes—sealed with muscle (type unspecified)
10 (Bajin et al., 2018)	N/A	Mondini	Cochleostomy	Oticon (NeuroEVO)	No	In the first month	N/A	N/A	Intravenous antibiotics	Yes—sealed with muscle (type unspecified)

The corresponding authors were contacted to obtain this information without success.

CSF indicates cerebrospinal fluid; IAC, internal auditory canal; N/A, information not provided.

The time from implantation to meningitis was recorded for 6 of 10 cases and ranged from 4 days to 30 months (Table 4). An intraoperative CSF leak was recorded for 6 of 10 cases of postoperative meningitis. All six cases of intraoperative CSF leak were suspected to be due to electrode insertion by the authors; other sources such as dural defects were not highlighted. Of the 10 cases of postoperative meningitis recorded, 6 received CIs without positioners; for the remaining 4, the positioner status was unrecorded (Table 4).

### Quality Assessment

All studies were evaluated through the National Institutes of Health Quality Assessment Tool. A full breakdown of the quality assessment per study is shown in Supplementary File 2 (http://links.lww.com/MAO/B657). Twenty-two studies were rated as having low risk of bias, 14 studies were rated as having moderate bias risk, and 2 studies were rated as having high bias risk.

## DISCUSSION

### Summary of Findings

The pooled proportion of meningitis after cochlear implantation in IEM was 0.12% (95% confidence interval, 0.006–0.380%). The little heterogeneity among studies (*I*^2^ = 0%) demonstrates the consistently low rate of meningitis across the included studies.

Although the incidence of postoperative meningitis in IEM implantation was extremely low (10 cases overall), cases occurred in patients with incomplete partition only (n = 5), Mondini deformity (n = 2), common cavity (n = 2), and enlarged IAC (n = 1). No cases were recorded in other tracked abnormalities, including cochlear hypoplasia, EVA, and incomplete partition combined with EVA. This may represent a random scattering of cases as there does not seem to be a correlation with degree of malformation, and the small case numbers prevented statistical analysis by IEM subtype.

### Comparison with Other Studies

The overall risk of meningitis following CI, including both normal and malformed cochleae, is difficult to estimate. There are relatively few large-scale series that report this risk, with most reporting a rate <0.4% (56,57). Reefhuis et al. (3) conducted a large epidemiologic study in the early 2000s, investigating postimplant meningitis risk in the pediatric population (<6 years). In this group, they showed that the incidence of postoperative meningitis was 239.3 per 100,000 person-years (95% confidence interval, 156.4–350.6). However, they did not report the number of patients with IEMs in their cohort and so could not calculate the rate of postimplant meningitis in this group. Instead, they used the 26 cases of postoperative meningitis encountered in their cohort to investigate risk factors by performing a nested case-control study. Using multivariate analysis, they found a statistically significant increased risk in those with an IEM and a concurrent intraoperative CSF leak (odds ratio, 9.3; 95% confidence interval, 1.2–94.5). Most cases of postoperative meningitis (6/10) in our meta-analysis were also associated with an intraoperative CSF leak.

An increased risk in IEMs after implantation could be due to abnormal labyrinthine architecture. Abnormal connections between the inner ear and the CSF-containing subarachnoid space, which are usually separate, can lead to a CSF leak when opening the cochlea during surgery (58). This can also allow a route for infection into the CSF. Two cases of reported meningitis involved incomplete partition type I. In this malformation, disruption of the interscalar septa and modiolus can result in a wide basal turn. A wide basal turn has been linked to a higher risk of CSF fistula formation and meningitis (9,59), but the numbers are too small to comment if this is specifically a higher risk malformation.

Incomplete partition also forms part of the Mondini deformity, along with dilated vestibule and EVA (7). This may allow a pathway for infection to the CSF through the bony canal housing the vestibular aqueduct. However, the term *Mondini* has been used to refer to a wide range of malformations (8).

One case of postoperative meningitis was reported in a patient with a wide IAC. The CSF in the subarachnoid space can extend laterally into fundus of the IAC, where the cribriform plate forms part of the barrier that separates this fluid from perilymph, and this is somewhat porous. This barrier can be disrupted with cochlear malformations, such as those affecting the IAC, allowing CSF and perilymph to mix (9,10). With the addition of a foreign body, such as a CI, this communication between the inner ear and CSF space can create a higher potential for otitic meningitis. However, there was no CSF leak reported in the IAC case with postoperative meningitis. Two patients with postoperative meningitis had a common cavity. In this malformation, the cochlea and vestibule are confluent, facilitating spread of a potential infection into the IAC and the subarachnoid space (60).

Four of 10 cases of postoperative meningitis were not associated with an intraoperative CSF leak, highlighting the role of other factors. Potential factors could include easier access to the CSF space than in normal ears if minor ingress of bacteria occurs because of more porous or thinner partition.

### Baseline Risk of Meningitis in Those with IEMs

It is important to note that patients with IEMs might be at higher risk of meningitis at baseline before any interventions (e.g., cochlear implantations). There have been several reports of sporadic meningitis in those with IEMs (61). Two studies in this systematic review reported patients with IEMs who had recurrent meningitis preoperatively but did not experience meningitis postoperatively (38,41). In addition, a case report has suggested the source of meningitis appeared to come from the nonimplanted ear in a patient with bilateral Mondini deformity (62). However, we did not identify any studies that attempted to measure or estimate the incidence of meningitis in those with IEMs (i.e., without any intervention). This information will be required, first, to see if those with IEMs are in fact at a higher risk of meningitis at baseline compared with the general population and, second, to see if there is an *additional* risk due to cochlear implantation in this population.

### Strengths and Limitations

This is the first study, to our knowledge, that systematically reports the postoperative meningitis rate after cochlear implantation in those with IEMs.

IEMs are not the only risk factors for postoperative meningitis. There are several other risk factors, including vaccination status, the use of a positioner (3), and young age, which could have also played a role. For instance, 18 of 38 studies reported a mean age at implantation of <5 years, and young age is an important risk factor for meningitis (63). To comment on the risk independently attributable to IEMs, the influence of these other risk factors needs to be analyzed through a multivariate analysis. However, no studies in this systematic review provided a breakdown of these potential factors, so this could not be performed.

All studies included in this review were observational. Individual case reports were not included, and adverse events databases (e.g., FDA Adverse Event Reporting System) were not searched. Although these sources provide the numerator (reports of meningitis), they do not provide the denominator (number of total implants placed) to calculate incidence. However, this approach might have led to some reports of postoperative meningitis being missed.

There were a notable number of IEMs that were not categorized (196/1300), which precluded an accurate subgroup meta-analysis by IEM type. However, the raw numbers of postoperative meningitis by subtype are included in Table 3.

## CONCLUSION

In those with IEMs, the risk of postoperative meningitis after cochlear implantation is low, with a low heterogeneity in rates across the studies included.

## Supplementary Material
