# Supplementary material for: Meningitis Risk in Patients with Inner Ear Malformations after Cochlear Implants: A Systematic Review and Meta-Analysis
Source: Otol Neurotol. 2023 Jun 15;44(7):627–35. doi: 10.1097/MAO.0000000000003913 (PMC10348644; doi:10.1097/MAO.0000000000003913)
Supplement: Supplementary file 1 [file on-44-0627-s001.docx]

Meningitis risk in patients with inner ear malformations after cochlear implants: A systematic review and meta-analysis.

Abstract

Objective:

To determine the rate of post-operative meningitis following cochlear implantation in those with inner ear malformations (IEMs) via meta-analysis.

Data sources:

Medline, EMBASE and the Cochrane Library.

Methods:

This study was reported following the preferred reporting items for systematic reviews and meta-analyses (PRISMA) checklist. Proportion meta-analysis was conducted through an inverse variance random-effect model based on arcsin transformation and presented as forest plots. Quality assessment of the included studies was performed through the NIH Quality Assessment Tool.

Results:

Overall, 38/2966 studies met the inclusion criteria and were included in the analysis. There were 10 cases of meningitis following cochlear implantation in 1300 malformed ears. The overall rate of meningitis after cochlear implantation in IEMs was 0.12% (95% confidence interval, 0.006-0.380%; I^2^=0%). Cases occurred in incomplete partition (n=5), Mondini deformity (n=2), common cavity (n=2), and enlarged internal auditory canal (n=1). Six out of ten cases of post-operative meningitis occurred with an intraoperative cerebrospinal fluid (CSF) leak.

Conclusion:

In those with IEMs, the risk of meningitis after cochlear implantation is very low.

Introduction

Cochlear implants (CIs) are surgically implanted devices that can improve hearing in those with severe to profound sensorineural hearing loss. By restoring sound perception, they also have wider effects on quality of life by reducing social isolation, anxiety and depression in implanted patients^1^.

However, as with any surgical intervention, there are potential complications associated with CIs. Minor complications often resolve spontaneously or with medical management, and range from dizziness to taste disturbance. Major complications may require revision surgery and/or hospitalisation, and include device migration, electrode extrusion and infections such as meningitis^2^.

Meningitis is a rare but life-threatening complication linked to CIs that has received a significant level of attention. This concern stemmed from a large epidemiological study in the early 2000s that found a higher risk of meningitis in those with CIs compared to the general population^3^. In those receiving cochlear implantation, proposed risk factors include CIs with intracochlear positioners. However, the cause for this is not entirely known with several etiologies being proposed, one of which includes positioner-induced modiolus trauma. Another proposed risk factor for post-operative meningitis includes the presence of inner ear malformations^3–5^.

Inner ear malformations (IEMs) can be classified into categories based on morphology, which may arise from developmental arrest, genetic abnormalities, or intrauterine factors^6,7^. Abnormalities during early development can lead to the cochlea being completely absent (*Cochlear aplasia)* or underdeveloped (*Cochlear Hypoplasia*). A cystic structure can result if differentiation between the cochlea and vestibule does not proceed (*Common Cavity).* Even if differentiation is successful, disruption of the central modiolus and/or interscalar septa can result in a cystic structure internally (*Incomplete Partition).* Other abnormalities include an *Enlarged Vestibular Aqueduct*, or dysplasia/ aplasia of the vestibular system and cochlear nerve. These malformations can also occur in combination, as is the case in *Mondini deformity.* This consists of the triad of incomplete partition II, dilated vestibule and enlarged vestibular aqueduct^7^. However, the term has been used to describe a range of other inner ear malformations^8^.

There are a range of reasons why IEMs could be at increased risk of meningitis. The presence of abnormal fistulae between the inner ear and subarachnoid space could allow communication and infection spread to the cerebrospinal fluid (CSF)^9^. Other reasons include malformations of the lamina cribrosa, which is a bony separation between the cochlea and internal auditory canal. This may be partially or completely absent and allow a route of infection from the middle ear to the inner ear and CSF space, leading to a higher potential for otogenic meningitis^10^. The cochlear implant can act as a nidus for infection, facilitating infection spread.

Many studies that have proposed a link between inner ear malformations and meningitis following cochlear implantation have been case reports or small case series, making it difficult to gauge true incidence and risk^11,12^. The aim of this study is to perform a systematic review and proportion meta-analysis of the literature to ascertain the rate of meningitis following CIs in those with IEMs.

Methods:

This systematic review and meta-analysis were performed in line with the preferred reporting items for systematic reviews and meta-analyses (PRISMA) guidelines^13^. This review was also registered on PROSPERO (Registration ID: CRD42022333508).

Aims

The aim was to perform a proportion meta-analysis of the prevalence of post-operative meningitis in patients with IEMs following cochlear implantation. The specific type of IEM implanted was recorded to determine whether a specific IEM was prone to a higher risk of meningitis.

Search strategy

The search strategy was designed with assistance from our University Medical librarian (IK). A search on Medline, Embase and the Cochrane Library was performed in January 2023 and combined word variants from 2 key themes: A) cochlear implants and B) inner ear malformations (Supplementary file 1, http://links.lww.com/MAO/B656). This led to 966 unique hits.

A second search was also performed combining ‘cochlear implantation’ and ‘complications’ (Supplementary File 1, http://links.lww.com/MAO/B656). This was designed to capture relevant studies that reported complications after implantation in a mixed population of patients: some with normal cochleae and others with IEMs. These studies fit the inclusion criteria as it was possible to determine whether meningitis, if reported, occurred in IEM or normal cochlea patients^14–16^. However, some of these studies were missed by the first search. This was potentially because they were only indexed under ‘cochlear implants’ and not under ‘IEMs’ as the latter were only a minor component of the paper. This search yielded 2000 unique hits.

Results from both searches were combined to increase comprehensiveness, yielding 2966 original articles. Two authors (SVG and AF) independently screened all the titles and abstracts resulting from the search, and then assessed the full texts of the relevant articles identified against the inclusion criteria. Disagreements were resolved through discussion. A third author (DB) resolved disagreements if discussion failed to reach a consensus. The references of all narrative reviews found were also screened to find relevant articles.

Inclusion and exclusion criteria

The inclusion criteria are presented through a PICOTS format in Table 1. The exclusion criteria included non-English language studies, case reports, editorials, letters, and reviews. Studies reporting other otological surgery at the same time as cochlear implantation were also excluded.

Data extraction

An electronic data collection form was used to collect the following information from included studies: author; year of publication; study design; number of patients with IEMs; gender breakdown; mean age at implantation; number of malformed ears implanted; radiological confirmation of IEMs with high resolution CT scan and/or MRI; types of malformed ears implanted; number of post-operative cases of meningitis; number of intra-operative CSF leak (including gusher); post-operative CSF leak and follow-up time (mean and range). The categories of inner ear malformations were recorded according to Sennaroglu’s version of the modified Jackler classification^6,7^, and included cochlear aplasia, cochlear hypoplasia, common cavity, incomplete partition of the cochlea (split into Types I, II and III if specified^7^), and enlarged vestibular aqueduct. Cochlear ossification was not included. Mondini deformity was recorded if the complete triad of incomplete partition II, minimally dilated vestibule and an enlarged vestibular aqueduct were present, or if the study reported Mondini dysplasia unspecified.

The data extraction form was designed by SVG, DB and MB. Two reviewers independently extracted the data and disagreements were resolved by a third reviewer. When relevant conference abstracts were identified, the authors were contacted to obtain data on the number of post-operative meningitis cases in their IEM cochlear implant cohort.

Statistical analysis

Proportion meta-analysis was conducted through an inverse variance random-effect model based on arcsin transformation and presented as forest plots. The weighted pooled proportion estimates and corresponding 95% confidence intervals (CIs) were calculated according to the random-effects models of DerSimonian and Laird^17^. For each study, proportions are depicted as grey squares, whereas relative 95% confidence interval (CI) as horizontal lines. The weight of each study on the overall effect estimate is reported and represented by the square size. The overall proportion estimates with relative 95% CIs are depicted as black diamonds at the bottom of the forest plot. Heterogeneity between studies was assessed with Higgins I^2^ and τ^2^ tests, defined as low if I^2^<25%, moderate if between 25-50%, and substantial if >50%^18^. Publication bias was assessed through funnel plots and Egger’s test^19,20^. Moderator analysis was conducted through subgroup analysis to assess whether the year of publication and number of patients included could be related to a higher risk of post-operative meningitis^19,21^. Statistical analysis was performed with R (version 4.2.1, R foundation for Statistical Computing, Vienna, Austria); packages ‘meta’ and ‘metafor’. Statistical significance was defined as p<0.05.

Risk of bias assessment

The National Institute for Health (NIH) quality assessment tool was used to analyse each study for risk of bias. Bias analysis was performed by two reviewers independently (SVG and AF), and disagreements were resolved through a third reviewer (DB).

Results:

Literature search

The first search specifying IEMs produced 966 unique hits. The second, broader search, with ‘cochlear implants’ and ‘complications’ produced 2000 unique hits. Reasons for these separate searches are outlined in the methods. The results of both searches were combined to yield 2966 unique hits. After screening these via title and abstract, 449 articles remained for full text screening. From this set, 38 articles were included in the final review after applying the inclusion and exclusion criteria. This is shown via a PRISMA flowchart in Figure 1

Background characteristics

The characteristics of all the included studies are outlined in Table 2^5,12,14–16,22–54^. All 38 studies were descriptive cohort studies without control^55^. Thirty-one studies provided the mean age at implantation. Thirty of these studies reported a mean age of < 18 years, with 18 of these 30 studies reporting a mean age of < 5 years. Six studies reported on the pneumococcal vaccination status of the population before implantation, confirming that all were vaccinated.

Number and breakdown of malformations

Cochlear implants were placed in a total of 1300 ears with IEMs. The most common malformation implanted was enlarged vestibular aqueduct (n = 332), followed by Mondini Deformity (n= 297) and incomplete partition of the cochlea only (n = 158). Incomplete partition in combination with enlarged vestibular aqueduct was recorded in 56 cases. The least common malformation was cochlear aplasia (n = 1). The underlying abnormality was not provided for 196 ears. A full breakdown by the type of malformation is given in Table 3. One hundred and thirty-one ears had other abnormalities outside the main categories of Sennaroglu’s classification^7^, such as semicircular canal dysplasia, and other vestibular defects.

Of note, 297 implanted ears had Mondini deformity. Of these, 217 were documented as having the triad of incomplete partition type 2, dilated vestibule, and EVA. We found that 80 ears were classified as Mondini’s, but the underlying deformities were not specified. Due to the discrepancies with this term, it is possible other abnormalities could have been classified as Mondini’s in these 80 cases.

Number of meningitis cases

A total of 10 cases of meningitis were recorded in 1300 ears implanted with inner ear malformations. The meta-analysis returned a rate of meningitis after CI in IEM of 0.12% (95% CI, 0.006-0.382%; I^2^=0%) (Figure 2). No publication bias was found with Egger’s test (p=0.515).

Cases were recorded in those with incomplete partition only (n=5), Mondini deformity (n=2), common cavity (n=2), and enlarged internal auditory canal (n=1). For each case of post-operative meningitis reported (n=10), available data on demographics, intraoperative factors such as electrode insertion technique, CSF leak, vaccination status and causative organism are provided in Table 4.

The time from implantation to meningitis was recorded for 6 out of 10 cases and ranged from 4 days to 30 months (Table 4). An intra-operative CSF leak was recorded for 6 out 10 cases of post-operative meningitis. All 6 cases of intraoperative CSF leak were suspected to be due to electrode insertion by the authors; other sources such as dural defects were not highlighted. Of the 10 cases of post-operative meningitis recorded, 6 received CIs without positioners; for the remaining 4 the positioner status was unrecorded (Table 4).

Quality assessment

All studies were evaluated through the NIH Quality Assessment tool. A full breakdown of the quality assessment per study is shown in Supplementary file 2, http://links.lww.com/MAO/B657. Twenty-two studies were rated as having low risk of bias, 14 studies were rated as having moderate bias risk, and 2 studies were rated as having high bias risk.

Discussion:

Summary of findings

The pooled proportion of meningitis after cochlear implantation in IEM was 0.12 % (95% CI, 0.006-0.380%). The little heterogeneity among studies (I^2^=0%) demonstrates the consistently low rate of meningitis across the included studies.

Although the incidence of post-operative meningitis in IEM implantation was extremely low (10 cases overall), cases occurred in patients with incomplete partition only (n=5), Mondini deformity (n=2), common cavity (n=2) and enlarged internal auditory canal (n=1). No cases were recorded in other tracked abnormalities including cochlear hypoplasia, EVA, and incomplete partition combined with EVA. This may represent a random scattering of cases as there does not seem to be a correlation with degree of malformation, and the small case numbers prevented statistical analysis by IEM subtype.

Comparison to other studies

The overall risk of meningitis following CI, including both normal and malformed cochleae, is difficult to estimate. There are relatively few large-scale series that report this risk, with most reporting a rate < 0.4%^56,57^. Reefhuis et al conducted a large epidemiological study in the early 2000s investigating post-implant meningitis risk in the paediatric population (<6 years)^3^. In this group, they showed that the incidence of post-operative meningitis was 239.3 per 100,000 person-years (95% confidence interval, 156.4 to 350.6). However, they did not report the number of patients with IEMs in their cohort, and so, could not calculate the rate of post-implant meningitis in this group. Instead, they used the 26 cases of post-operative meningitis encountered in their cohort to investigate risk factors by performing a nested case-control study. Using multi-variate analysis, they found a statistically significant increased risk in those with an IEM and a concurrent intraoperative CSF leak (odds ratio: 9.3; 95 percent confidence interval, 1.2 to 94.5). Most cases of post-operative meningitis (6/10) in our meta-analysis were also associated with an intra-operative CSF leak.

An increased risk in IEMs following implantation could be due to abnormal labyrinthine architecture. Abnormal connections between the inner ear and the CSF-containing subarachnoid space, which are usually separate, can lead to a CSF leak when opening the cochlea during surgery^58^. This can also allow a route for infection into the CSF. Two cases of reported meningitis involved incomplete partition type I. In this malformation, disruption of the interscalar septa and modiolus can result in a wide basal turn. A wide basal turn has been linked to a higher risk of CSF fistula formation and meningitis^9,59^, but the numbers are too small to comment if this is specifically a higher risk malformation.

Incomplete partition also forms part of the Mondini deformity, along with dilated vestibule and enlarged vestibular aqueduct^7^. This may allow a pathway for infection to the CSF through the bony canal housing the vestibular aqueduct. However, the term Mondini has been used to refer to a wide range of malformations^8^.

One case of post-operative meningitis was reported in a patient with a wide internal auditory canal (IAC). The CSF in the subarachnoid space can extend laterally into fundus of the IAC, where the cribriform plate forms part of the barrier that separates this fluid from perilymph, and this is somewhat porous. This barrier can be disrupted with cochlear malformations, such as those affecting the IAC, allowing CSF and perilymph to mix^9,10^. With the addition of a foreign body, such as a cochlear implant, this communication between the inner ear and CSF space can create a higher potential for otitic meningitis. However, there was no CSF leak reported in the IAC case with post-operative meningitis. Two patients with post-operative meningitis had a common cavity. In this malformation, the cochlea and vestibule are confluent, facilitating spread of a potential infection into the internal auditory canal and the subarachnoid space^60^.

Four out of ten cases of post-operative meningitis were not associated with an intra-operative CSF leak, highlighting the role of other factors. Potential factors could include easier access to the CSF space than in normal ears if minor ingress of bacteria occurs because of more porous or thinner partition.

Baseline risk of meningitis in those with IEMs

It is important to note that patients with inner ear malformations might be at higher risk of meningitis at baseline prior to any interventions (e.g, cochlear implantations). There have been several reports of sporadic meningitis in those with IEMs^61^. Two studies in this systematic review reported patients with IEMs who had recurrent meningitis pre-operatively, but did not experience meningitis post-operatively^38,41^. In addition, a case report has suggested the source of meningitis appeared to come from the non-implanted ear in a patient with bilateral Mondini’s deformity^62^. However, we did not identify any studies that attempted to measure or estimate the incidence of meningitis in those with IEMs (i.e., without any intervention). This information will be required firstly to see if those with IEMs are in fact at a higher risk of meningitis at baseline compared to the general population, and secondly, to see if there is an *additional* risk due to cochlear implantation in this population.

Strengths and limitations

This is the first study, to our knowledge, that systematically reports the post-operative meningitis rate following cochlear implantation in those with inner ear malformations.

Inner ear malformations are not the only risk factors for post-operative meningitis. There are several other risk factors, including vaccination status, the use of a positioner^3^, and young age, which could have also played a role. For instance, 18/38 studies reported a mean age at implantation of < 5 years, and young age is an important risk factor for meningitis^63^. In order to comment on the risk independently attributable to IEMs, the influence of these other risk factors needs to be analysed through a multi-variate analysis. However, no studies in this systematic review provided a breakdown of these potential factors, so this could not be performed.

All studies included in this review were observational. Individual case reports were not included and adverse events databases (e.g, FDA Adverse Event Reporting System) were not searched. While these sources provide the numerator (reports of meningitis), they do not provide the denominator (number of total implants placed) to calculate incidence. However, this approach might have led to some reports of post-operative meningitis being missed.

There were a notable number of IEMs that were not categorised (196/1300), which precluded an accurate subgroup meta-analysis by IEM type. However, the raw numbers of post-operative meningitis by subtype are included in Table 3.

Conclusion

In those with inner ear malformations, the risk of post-operative meningitis following cochlear implantation is low, with a low heterogeneity in rates across the studies included.

References

1. Mo B, Lindbaek M, Harris S. Cochlear implants and quality of life: a prospective study. *Ear Hear*. 2005;26(2):186-194. doi:10.1097/00003446-200504000-00006

2. Cohen NL, Hoffman RA. Complications of cochlear implant surgery in adults and children. *Annals of Otology, Rhinology & Laryngology*. 1991;100(9):708-711.

3. Reefhuis J, Honein MA, Whitney CG, et al. Risk of bacterial meningitis in children with cochlear implants. *New England Journal of Medicine*. 2003;349(5):435-445.

4. Cohen N, Ramos A, Ramsden R, et al. International consensus on meningitis and cochlear implants. *Acta Otolaryngol*. 2005;125(9):916-917. doi:10.1080/00016480510044403

5. Theunisse HJ, Pennings RJE, Kunst HPM, Mulder JJ, Mylanus EAM. Risk factors for complications in cochlear implant surgery. *European Archives of Oto-Rhino-Laryngology*. 2018;275(4):895-903.

6. Jackler RK, Luxford WM, House WF. Congenital malformations of the inner ear: a classification based on embryogenesis. *Laryngoscope*. 1987;97(3 Pt 2 Suppl 40):2-14. doi:10.1002/lary.5540971301

7. Sennaroğlu L, Demir Bajin M. Classification and Current Management of Inner Ear Malformations. *Balkan Med J*. 2017;34(5):397-411. doi:10.4274/balkanmedj.2017.0367

8. Lo WWM. What is a ‘Mondini’ and What Difference Does a Name Make? *American Journal of Neuroradiology*. 1999;20(8):1442-1444.

9. Phelps PD, King A, Michaels L. Cochlear dysplasia and meningitis. *Am J Otol*. 1994;15(4):551-557.

10. Papsin BC. Cochlear Implantation in Children With Anomalous Cochleovestibular Anatomy. *The Laryngoscope*. 2005;115(S106):1-26. doi:10.1097/00005537-200501001-00001

11. Page EL, Eby TL. Meningitis after cochlear implantation in Mondini malformation. *Otolaryngol Head Neck Surg*. 1997;116(1):104-106. doi:10.1016/s0194-5998(97)70358-9

12. Ahn JH, Chung JW, Lee KS. Complications following cochlear implantation in patients with anomalous inner ears: experiences in Asan Medical Center. *Acta Oto-Laryngologica*. 2008;128(1):38-42.

13. Welch V, Petticrew M, Tugwell P, et al. PRISMA-Equity 2012 Extension: Reporting Guidelines for Systematic Reviews with a Focus on Health Equity. *PLOS Medicine*. 2012;9(10):e1001333. doi:10.1371/journal.pmed.1001333

14. Gysin C, Papsin BC, Daya H, Nedzelski J. Surgical outcome after paediatric cochlear implantation: diminution of complications with the evolution of new surgical techniques. *Journal of Otolaryngology*. 2000;29(5):285-289.

15. Tarkan O, Tuncer U, Ozdemir S, et al. Surgical and medical management for complications in 475 consecutive pediatric cochlear implantations. *International Journal of Pediatric Otorhinolaryngology*. 2013;77(4):473-479.

16. Yang Y, Chen M, Zheng J, et al. Clinical evaluation of cochlear implantation in children younger than 12 months of age. *Pediatric Investigation*. 2020;4(2):99-103.

17. DerSimonian R, Laird N. Meta-analysis in clinical trials. *Control Clin Trials*. 1986;7(3):177-188. doi:10.1016/0197-2456(86)90046-2

18. Higgins JPT, Thompson SG, Deeks JJ, Altman DG. Measuring inconsistency in meta-analyses. *BMJ*. 2003;327(7414):557-560. doi:10.1136/bmj.327.7414.557

19. Wang N. *How to Conduct a Meta-Analysis of Proportions in R: A Comprehensive Tutorial*.; 2018. doi:10.13140/RG.2.2.27199.00161

20. Viechtbauer W. Conducting Meta-Analyses in R with the metafor Package. *Journal of Statistical Software*. 2010;36:1-48. doi:10.18637/jss.v036.i03

21. Thompson SG, Higgins JPT. How should meta-regression analyses be undertaken and interpreted? *Stat Med*. 2002;21(11):1559-1573. doi:10.1002/sim.1187

22. Ahn JH, Lim HW, Lee KS. Hearing improvement after cochlear implantation in common cavity malformed cochleae: long-term follow-up results. *Acta Oto-Laryngologica*. 2011;131(9):908-913.

23. Bae SH, Choi J, Choi JY. Cochlear Implants for Patients With Common Cavity Deformities and the Impact of Electrode Positioning. *Clin Exp Otorhinolaryngol*. 2022;15(1):77-83. doi:10.21053/ceo.2021.00745

24. Bajin MD, Pamuk AE, Pamuk G, Özgen B, Sennaroğlu L. The Association Between Modiolar Base Anomalies and Intraoperative Cerebrospinal Fluid Leakage in Patients With Incomplete Partition Type-II Anomaly: A Classification System and Presentation of 73 Cases. *Otol Neurotol*. 2018;39(7):e538-e542. doi:10.1097/MAO.0000000000001871

25. Beltrame MA, Birman CS, Cervera Escario J, et al. Common cavity and custom-made electrodes: speech perception and audiological performance of children with common cavity implanted with a custom-made MED-EL electrode. *International Journal of Pediatric Otorhinolaryngology*. 2013;77(8):1237-1243.

26. Bent JP, Chute P, Parisier SC. Cochlear implantation in children with enlarged vestibular aqueducts. *Laryngoscope*. 1999;109(7 Pt 1):1019-1022. doi:10.1097/00005537-199907000-00001

27. BERRETTINI S, FORLI F, DE VITO A, BRUSCHINI L, QUARANTA N. Cochlear implant in incomplete partition type I. *Acta Otorhinolaryngol Ital*. 2013;33(1):56-62.

28. Eftekharian A, Eftekharian K, Mokari N, Fazel M. Cochlear implantation in incomplete partition type I. *European Archives of Oto-Rhino-Laryngology*. 2019;276(10):2763-2768.

29. Grover M, Sharma S, Samdani S, et al. New SMS Classification of Cochleovestibular Anomalies: Our Experience with 25 Cases of Type I Anomaly. *Indian Journal of Otolaryngology & Head & Neck Surgery*. 2021;73(3):333-339.

30. Halawani R, Alzhrani F, Almuhawas F, Hagr AA. FORM24 electrode array and perioperative cerebrospinal fluid leakage in cochlear implant recipients with cochleovestibular malformations. *Annals of Saudi Medicine*. 2020;40(6):477-481.

31. Kim LS, Jeong SW, Huh MJ, Park YD. Cochlear implantation in children with inner ear malformations. *Annals of Otology, Rhinology & Laryngology*. 2006;115(3):205-214.

32. Kontorinis G, Goetz F, Giourgas A, Lenarz T, Lanfermann H, Giesemann AM. Radiological diagnosis of incomplete partition type I versus type II: significance for cochlear implantation. *European Radiology*. 2012;22(3):525-532.

33. Lai R, Hu P, Zhu F, et al. Genetic diagnosis and cochlear implantation for patients with nonsyndromic hearing loss and enlarged vestibular aqueduct. *J Laryngol Otol*. 2012;126(4):349-355. doi:10.1017/S002221511100346X

34. Lee KH, Lee J, Isaacson B, Kutz JW, Roland PS. Cochlear implantation in children with enlarged vestibular aqueduct. *Laryngoscope*. 2010;120(8):1675-1681.

35. Lescanne E, Al Zahrani M, Bakhos D, Robier A, Moriniere S. Revision surgeries and medical interventions in young cochlear implant recipients. *International Journal of Pediatric Otorhinolaryngology*. 2011;75(10):1221-1224.

36. Li S, Qin Z, Zhang F, Li L, Qi S, Liu L. Early complications following cochlear implantation in children and their management. *International Journal of Pediatric Otorhinolaryngology*. 2014;78(7):1040-1044.

37. Loundon N, Leboulanger N, Maillet J, et al. Cochlear implant and inner ear malformation. Proposal for an hyperosmolar therapy at surgery. *International Journal of Pediatric Otorhinolaryngology*. 2008;72(4):541-547.

38. Luntz M, Balkany T, Hodges AV, Telischi FF. Cochlear implants in children with congenital inner ear malformations. *Archives of Otolaryngology – Head & Neck Surgery*. 1997;123(9):974-977.

39. Manzoor NF, Wick CC, Wahba M, et al. Bilateral Sequential Cochlear Implantation in Patients With Enlarged Vestibular Aqueduct (EVA) Syndrome. *Otology & Neurotology*. 2016;37(2):e96-103.

40. Mey K, Bille M, Caye-Thomasen P. Cochlear implantation in Pendred syndrome and non-syndromic enlarged vestibular aqueduct - clinical challenges, surgical results, and complications. *Acta Oto-Laryngologica*. 2016;136(10):1064-1068.

41. Mylanus EA, Rotteveel LJ, Leeuw RL. Congenital malformation of the inner ear and pediatric cochlear implantation. *Otology & Neurotology*. 2004;25(3):308-317.

42. Pradhananga RB, Thomas JK, Natarajan K, Kameswaran M. Long term outcome of cochlear implantation in five children with common cavity deformity. *Int J Pediatr Otorhinolaryngol*. 2015;79(5):685-689. doi:10.1016/j.ijporl.2015.02.015

43. Qi S, Kong Y, Xu T, et al. Speech development in young children with Mondini dysplasia who had undergone cochlear implantation. *International Journal of Pediatric Otorhinolaryngology*. 2019;116:118-124. doi:10.1016/j.ijporl.2018.10.013

44. Rachovitsas D, Psillas G, Chatzigiannakidou V, Triaridis S, Constantinidis J, Vital V. Speech perception and production in children with inner ear malformations after cochlear implantation. *Int J Pediatr Otorhinolaryngol*. 2012;76(9):1370-1374. doi:10.1016/j.ijporl.2012.06.009

45. Sharma S, Grover M, Samdani S, Gupta G, Preetam C. SMS classification of inner ear malformations: our experience with implantation in type II anomalies. *European Archives of Oto Rhino Laryngology*. 2021;20:20.

46. Smeds H, Wales J, Asp F, et al. X-linked Malformation and Cochlear Implantation. *Otology & Neurotology*. 2017;38(1):38-46.

47. Suk Y, Lee JH, Lee KS. Surgical outcomes after cochlear implantation in children with incomplete partition type I: comparison with deaf children with a normal inner ear structure. *Otol Neurotol*. 2015;36(1):e11-17. doi:10.1097/MAO.0000000000000606

48. Suri NM, Prasad AR, Sayani RK, Anand A, Jaychandran G. Cochlear implantation in children with Mondini dysplasia: our experience. *J Laryngol Otol*. 2021;135(2):125-129. doi:10.1017/S0022215121000372

49. Tay SY, Anicete R, Tan KKH. A Ten-Year Review of Audiological Performance in Children with Inner Ear Abnormalities after Cochlear Implantation in Singapore. *Int J Otolaryngol*. 2019;2019:6483714. doi:10.1155/2019/6483714

50. Tian H, Wang L, Gao F, Liang W, Peng KA. Cochlear implantation using a custom guide catheter in 14 patients with incomplete partition type III. *Clin Otolaryngol*. 2018;43(5):1379-1383. doi:10.1111/coa.13146

51. Van Wermeskerken GK, Dunnebier EA, Van Olphen AF, Van Zanten BA, Albers FW. Audiological performance after cochlear implantation: a 2-year follow-up in children with inner ear malformations. *Acta Oto-Laryngologica*. 2007;127(3):252-257.

52. Wei X, Li Y, Fu QJ, et al. Slotted labyrinthotomy approach with customized electrode for patients with common cavity deformity. *Laryngoscope*. 2018;128(2):468-472.

53. Xia J, Wang W, Zhang D. Cochlear implantation in 21 patients with common cavity malformation. *Acta Otolaryngol*. 2015;135(5):459-465. doi:10.3109/00016489.2014.990054

54. Ding X, Tian H, Wang W, Zhang D. Cochlear implantation in China: review of 1,237 cases with an emphasis on complications. *Orl; Journal of Oto-Rhino-Laryngology & its Related Specialties*. 2009;71(4):192-195.

55. Dekkers OM, Egger M, Altman DG, Vandenbroucke JP. Distinguishing case series from cohort studies. *Ann Intern Med*. 2012;156(1 Pt 1):37-40. doi:10.7326/0003-4819-156-1-201201030-00006

56. Ovesen T, Johansen LV. Post-operative problems and complications in 313 consecutive cochlear implantations. *Journal of Laryngology & Otology*. 2009;123(5):492-496.

57. Farinetti A, Ben Gharbia D, Mancini J, Roman S, Nicollas R, Triglia JM. Cochlear implant complications in 403 patients: comparative study of adults and children and review of the literature. *European annals of otorhinolaryngology, head & neck diseases*. 2014;131(3):177-182.

58. Miyamoto RT, McConkey Robbins AJ, Myres WA, Pope ML. COCHLEAR IMPLANTATION IN THE MONDINI INNER EAR MALFORMATION. *Otology & Neurotology*. 1986;7(4):258.

59. Phelps PD, Proops D, Sellars S, Evans J, Michaels L. Congenital cerebrospinal fluid fistula through the inner ear and meningitis. *The Journal of Laryngology & Otology*. 1993;107(6):492-495. doi:10.1017/S0022215100123552

60. Phelps PD. The common cavity deformity of the ear. A precursor of meningitis but now being implanted. *JBR-BTR*. 1999;82(5):239-240.

61. Muzzi E, Battelino S, Gregori M, Pellegrin A, Orzan E. Life-threatening unilateral hearing impairments. Review of the literature on the association between inner ear malformations and meningitis. *International Journal of Pediatric Otorhinolaryngology*. 2015;79(12):1969-1974. doi:10.1016/j.ijporl.2015.09.028

62. Suzuki C, Sando I, Fagan JJ, Kamerer DB, Knisely AS. Histopathological features of a cochlear implant and otogenic meningitis in Mondini dysplasia. *Arch Otolaryngol Head Neck Surg*. 1998;124(4):462-466. doi:10.1001/archotol.124.4.462

63. Dickinson FO, Pérez AE. Bacterial Meningitis in children and adolescents: an observational study based on the national surveillance system. *BMC Infectious Diseases*. 2005;5(1):103. doi:10.1186/1471-2334-5-103

Legends and headings

Figure 1: PRISMA flowchart of the search and screening process

Figure 2: Forest plot for studies reporting the rate of post-implant meningitis in those with inner ear malformations

Table 1: Description of the study design using the PICOTS format. This also contains the inclusion criteria for the study.

Table 2: Characteristics of included studies

Table 3: Breakdown of malformed ears by the type of inner ear malformation, and the number of post-operative meningitis cases by type of inner ear malformation.

Table 4: Characteristics of those with post-operative meningitis
