# Supplementary material for: Meningitis Risk in Patients with Inner Ear Malformations after Cochlear Implants: A Systematic Review and Meta-Analysis
Source: Otol Neurotol. 2023 Jun 15;44(7):627–35. doi: 10.1097/MAO.0000000000003913 (PMC10348644; doi:10.1097/MAO.0000000000003913)
Supplement: Supplementary file 2 [file on-44-0627-s002.docx]

Supplementary file 1: Full search strategy for the systematic review.

Search A:

Embase

Embase <1974 to 2023 January 20>

1 exp *inner ear malformation/ 740

2 exp *Pendred syndrome/ 302

3 (common cavity or cochlear aplasia or cochlear hypoplasia or incomplete partition* or enlarged vestibular aqueduct* or Mondini or ((inner ear* or middle ear* or bony labyrinth* or membranous labyrinth* or cochleovestibular or vestibular) adj3 (malform* or anomal* or deform* or abnormal* or dyplasi*)) or pendred* or large vestibular aqueduct syndrome* or eva or lvas or (warsaw adj3 syndrome*)).ti,ab. 8525

4 1 or 2 or 3 8762

5 (cochlea* adj5 (implant* or prosthe* or devic*)).ti,ab. 20611

6 exp *cochlea prosthesis/ 12395

7 exp *cochlear implantation/ 3477

8 5 or 6 or 7 21561

9 4 and 8 823

Medline

Ovid MEDLINE(R) and Epub Ahead of Print, In-Process, In-Data-Review & Other Non-Indexed Citations, Daily and Versions <1946 to January 20, 2023>

1 (common cavity or cochlear aplasia or cochlear hypoplasia or incomplete partition* or enlarged vestibular aqueduct* or Mondini or ((inner ear* or bony labyrinth* or membranous labyrinth* or cochleovestibular or vestibular) adj3 (malform* or anomal* or deform* or abnormal* or dyplasi*)) or pendred* or large vestibular aqueduct syndrome* or eva or lvas or (warsaw adj3 syndrome*)).ti,ab,kw,kf. 6690

2 (cochlea* adj5 (implant* or prosthe* or devic*)).ti,ab,kw,kf. 18030

3 exp Cochlear Implants/ 12326

4 exp Cochlear Implantation/ 8916

5 2 or 3 or 4 19632

6 1 and 5 718

Cochrane Library

Search Name:

Date Run: 23/01/2023 17:33:02

Comment:

ID Search Hits

#1 (cochlea* near5 (implant* or prosthe* or devic*)) 29

#2 MeSH descriptor: [Cochlear Implants] explode all trees 163

#3 MeSH descriptor: [Cochlear Implantation] explode all trees 99

#4 #1 or #2 or #3 220

#5 cochlear implants 341

#6 cochlear prosthesis 162

#7 cochlear prosthetic 8

#8 cochlear device 121

#9 #4 or #5 or #6 or #7 or #8 507

#10 common cavity or cochlear aplasia or cochlear hypoplasia or incomplete partition* or enlarged vestibular aqueduct* or Mondini 1548

#11 ((inner ear* or middle ear* or bony labyrinth* or membranous labyrinth* or cochleovestibular) near (malform* or anomal* or deform* or abnormal*)) 803

#12 pendred or large vestibular aqueduct syndrome* or eva or lvas 1442

#13 warsaw syndrome 92

#14 #10 or #11 or #12 or #13 3825

#15 #4 and #14 12

Summary of Search A:

|  | **Jan 2023** |
| --- | --- |
| Medline via Ovid | 718 |
| Embase via Ovid | 823 |
| Cochrane Library | 12 |
| **Total** | **1553** |
| **Total Deduplicated** | **966** |

Search B

Embase

Embase <1974 to 2023 January 23>

1 (cochlea* adj5 (implant* or prosthe* or devic*)).ti,ab.

2 exp *cochlea prosthesis/

3 exp *cochlear implantation/

4 1 or 2 or 3

5 (((post-operat* or post-surg* or postoperat* or postsurg* or post operat* or post surg*) adj3 complicat*) or ((post-operat* or post-surg* or postoperat* or postsurg* or post operat* or post surg*) adj3 infect*)).ti,ab.

6 exp *postoperative complication/

7 ("escherichia coli" or "e coli").ti,ab.

8 "listeria monocytogenes".ti,ab.

9 ((haemophilus or hemophilus or hib) adj2 (mening* or infect*)).ti,ab.

10 ("streptococcus group b" or "streptococcus agalactiae").ti,ab.

11 (meningit* or meningococ*).ti,ab.

12 exp *meningitis/ or exp *bacterial meningitis/

13 exp *meningococcosis/

14 exp *pneumococcal infection/

15 exp *Neisseria meningitidis/

16 exp *Escherichia coli infection/

17 exp *Listeria monocytogenes/

18 exp *Listeria meningitis/

19 exp *Haemophilus influenzae type b/

20 exp *Haemophilus infection/

21 exp *Haemophilus meningitis/

22 exp *Streptococcus agalactiae/

23 or/5-22

24 4 and 23 1113

Medline

Ovid MEDLINE(R) and Epub Ahead of Print, In-Process, In-Data-Review & Other Non-Indexed Citations, Daily and Versions(R) <1946 to January 23, 2023>

1 (cochlea* adj5 (implant* or prosthe* or devic*)).ti,ab,kw,kf.

2 exp Cochlear Implants/

3 exp Cochlear Implantation/

4 1 or 2 or 3

5 (((post-operat* or post-surg* or postoperat* or postsurg* or post operat* or post surg*) adj3 complicat*) or ((post-operat* or post-surg* or postoperat* or postsurg* or post operat* or post surg*) adj3 infect*)).ti,ab,kw,kf.

6 exp Postoperative Complications/

7 Meningitis/ or exp Meningitis, Bacterial/ or exp Meningococcal Infections/ or exp Pneumococcal Infections/ or exp Neisseria meningitidis/ or exp Escherichia coli Infections/ or ("escherichia coli" or "e coli").ti,ab,kw,kf. or (Listeria monocytogenes/ or Meningitis, Listeria/) or "listeria monocytogenes".ti,ab,kw,kf. or Haemophilus influenzae type b/ or (haemophilus infections/ or meningitis, haemophilus/) or ((haemophilus or hemophilus or hib) adj2 (mening* or infect*)).ti,ab,kw,kf. or Streptococcus agalactiae/ or ("streptococcus group b" or "streptococcus agalactiae").ti,ab,kw,kf. or (meningit* or meningococ*).ti,ab,kw,kf.

8 5 or 6 or 7

9 4 and 8 1619

Cochrane Library

Search Name:

Date Run: 23/01/2023

Comment:

ID Search Hits

#1 (cochlea* near5 (implant* or prosthe* or devic*))

#2 MeSH descriptor: [Cochlear Implants] explode all trees

#3 MeSH descriptor: [Cochlear Implantation] explode all trees

#4 #1 or #2 or #3

#5 cochlear implants

#6 cochlear prosthesis

#7 cochlear prosthetic

#8 cochlear device

#9 #4 or #5 or #6 or #7 or #8

#10 ((post-operat* or post-surg* or postoperat* or postsurg* or post operat* or post surg*) near complicat*):ti,ab,kw (Word variations have been searched)

#11 ((post-operat* or post-surg* or postoperat* or postsurg* or post operat* or post surg*) near infect*):ti,ab,kw (Word variations have been searched)

#12 MeSH descriptor: [Postoperative Complications] explode all trees

#13 MeSH descriptor: [Meninges] explode all trees

#14 MeSH descriptor: [Meningitis, Bacterial] explode all trees

#15 MeSH descriptor: [Meningococcal Infections] explode all trees

#16 MeSH descriptor: [Pneumococcal Infections] explode all trees

#17 MeSH descriptor: [Neisseria meningitidis] explode all trees

#18 MeSH descriptor: [Escherichia coli Infections] explode all trees

#19 MeSH descriptor: [Listeria monocytogenes] explode all trees

#20 MeSH descriptor: [Meningitis, Listeria] explode all trees

#21 MeSH descriptor: [Haemophilus influenzae type b] explode all trees

#22 MeSH descriptor: [Haemophilus Infections] explode all trees

#23 MeSH descriptor: [Meningitis, Haemophilus] explode all trees

#24 MeSH descriptor: [Streptococcus agalactiae] explode all trees

#25 ("escherichia coli" or "e coli")

#26 "listeria monocytogenes"

#27 (haemophilus or hemophilus or hib) near (mening* or infect*)

#28 ("streptococcus group b" or "streptococcus agalactiae")

#29 meningit* or meningococ*

#30 #10 or #11 or #12 or #13 or #14 or #15 or #16 or #17 or #18 or #19 or #20 or #21 or #22 or #23 or #24 or #25 or #26 or #27 or #28 or #29 95184

#31 #9 and #30 35

Summary Search B:

|  | **Jan 2023** |
| --- | --- |
| Medline via Ovid | 1619 |
| Embase via Ovid | 1113 |
| Cochrane Library | 35 |
| **Total** | **2767** |
| **Total Deduplicated** | **2000** |

Combining Search A and B:

|  | **Search A** | **Search B** | **Combined** |
| --- | --- | --- | --- |
| Medline via Ovid | 718 | 1619 | 2337 |
| Embase via Ovid | 823 | 1113 | 1936 |
| Cochrane Library | 12 | 35 | 47 |
| **Total** | **1553** | **2767** | **4320** |
| **Total Deduplicated** | **966** | **2000** | **2966** |
