# Supplementary material for: Meningitis Risk in Patients with Inner Ear Malformations after Cochlear Implants: A Systematic Review and Meta-Analysis
Source: Otol Neurotol. 2023 Jun 15;44(7):627–35. doi: 10.1097/MAO.0000000000003913 (PMC10348644; doi:10.1097/MAO.0000000000003913)

Supplementary file 2: Risk of bias analysis of the included papers performed through the National Institute of Health Quality Assessment Tool


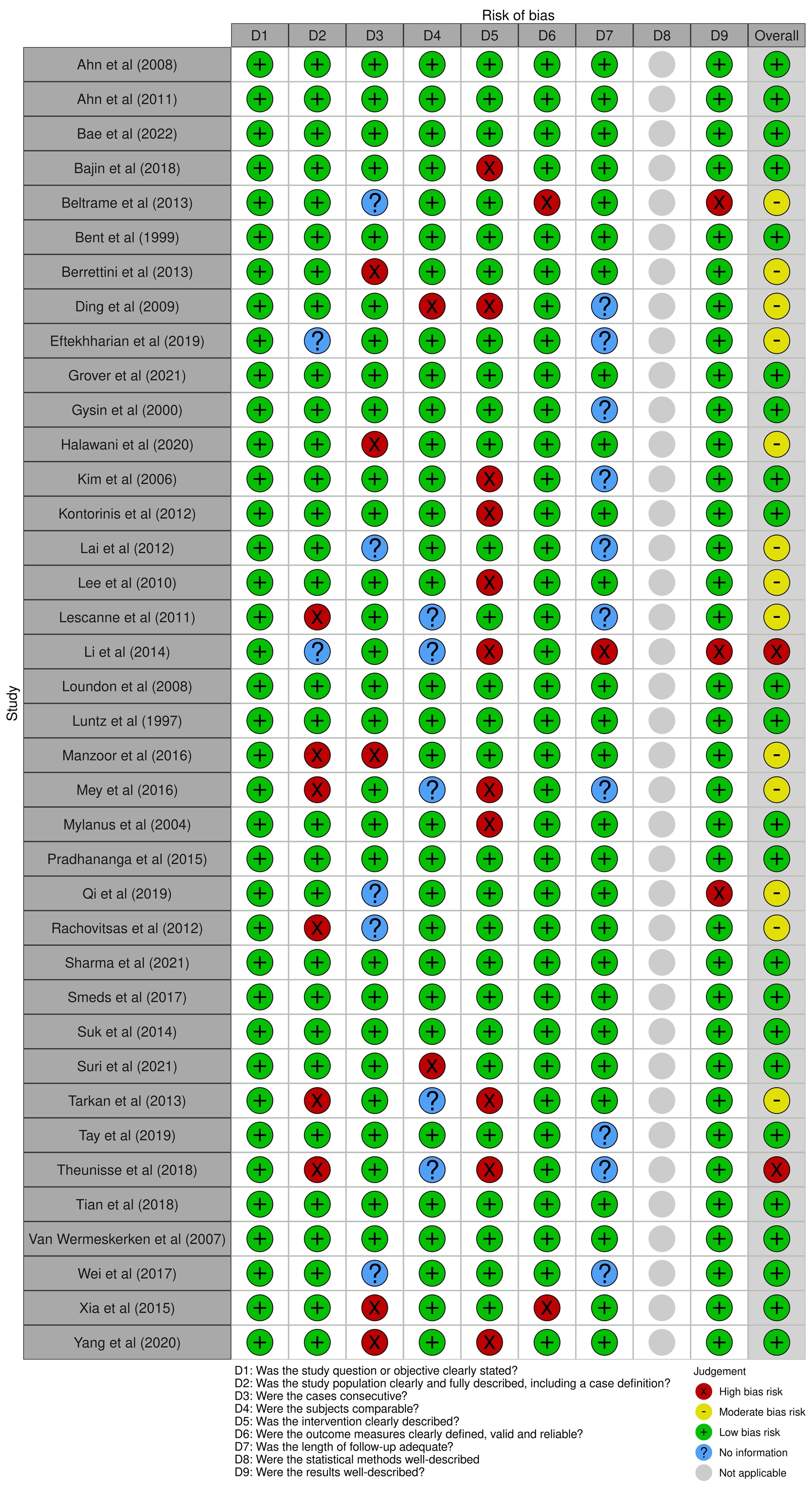


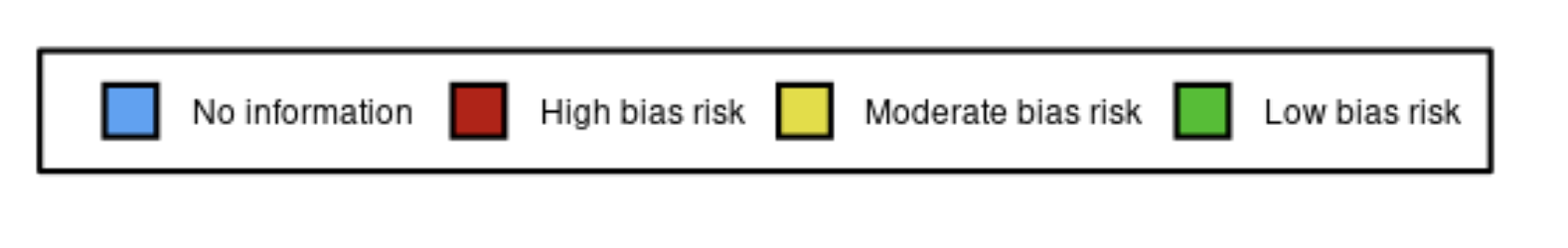

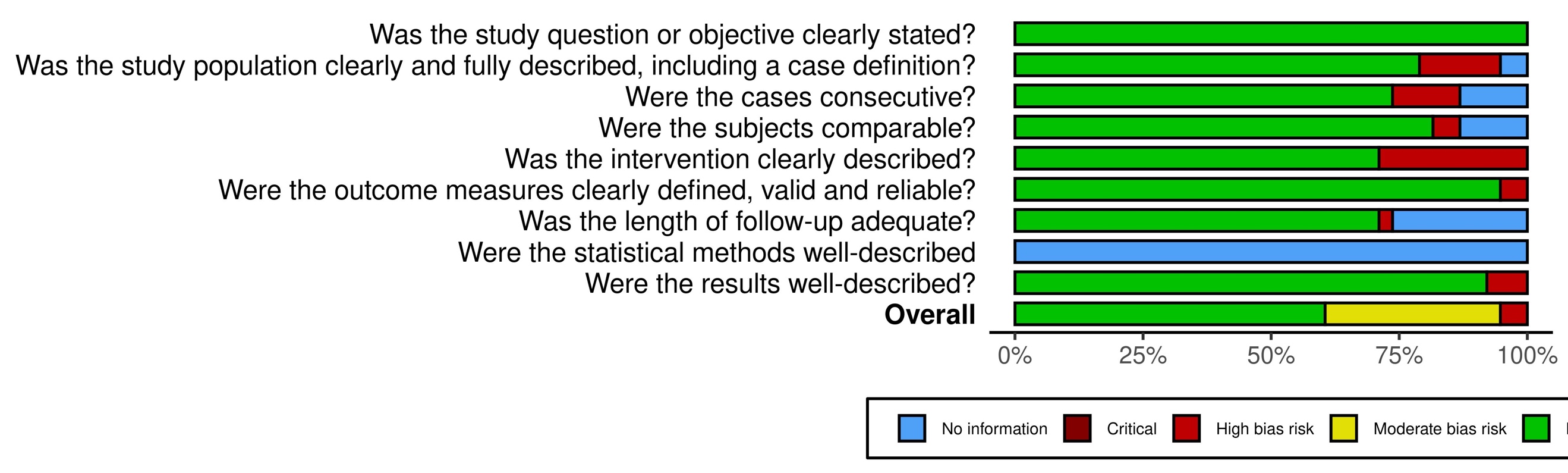

Supplement: Supplementary file 3 [file on-44-0627-s003.docx]
